# Urine Arsenic Concentrations and Species Excretion Patterns in American Indian Communities Over a 10-year Period: The Strong Heart Study

**DOI:** 10.1289/ehp.0800509

**Published:** 2009-05-07

**Authors:** Ana Navas-Acien, Jason G. Umans, Barbara V. Howard, Walter Goessler, Kevin A. Francesconi, Ciprian M. Crainiceanu, Ellen K. Silbergeld, Eliseo Guallar

**Affiliations:** 1 Department of Environmental Health Sciences, Johns Hopkins Bloomberg School of Public Health, Baltimore, Maryland, USA; 2 Department of Epidemiology and Welch Center for Prevention, Epidemiology, and Clinical Research, Johns Hopkins Medical Institutions, Baltimore, Maryland, USA; 3 MedStar Research Institute, Washington, DC, USA; 4 Institute of Chemistry–Analytical Chemistry, Karl-Franzens University Graz, Graz, Austria; 5 Department of Biostatistics, Johns Hopkins Bloomberg School of Public Health, Baltimore, Maryland, USA; 6 Department of Cardiovascular Epidemiology and Population Genetics, Centro Nacional de Investigaciones Cardiovasculares, Madrid, Spain

**Keywords:** American Indians, analytical chemistry, arsenic, arsenic species, arsenobetaine, exposure assessment, metabolism, mixed-effects models, multilevel analysis, Strong Heart Study

## Abstract

**Background:**

Arsenic exposure in drinking water disproportionately affects small communities in some U.S. regions, including American Indian communities. In U.S. adults with no seafood intake, median total urine arsenic is 3.4 μg/L.

**Objective:**

We evaluated arsenic exposure and excretion patterns using urine samples collected over 10 years in a random sample of American Indians from Arizona, Oklahoma, and North and South Dakota who participated in a cohort study from 1989 to 1999.

**Methods:**

We measured total urine arsenic and arsenic species [inorganic arsenic (arsenite and arsenate), methylarsonate (MA), dimethylarsinate (DMA), and arsenobetaine] concentrations in 60 participants (three urine samples each, for a total of 180 urine samples) using inductively coupled plasma/mass spectrometry (ICPMS) and high-performance liquid chromatography/ICPMS, respectively.

**Results:**

Median (10th, 90th percentiles) urine concentration for the sum of inorganic arsenic, MA, and DMA at baseline was 7.2 (3.1, 16.9) μg/g creatinine; the median was higher in Arizona (12.5 μg/g), intermediate in the Dakotas (9.1 μg/g), and lower in Oklahoma (4.4 μg/g). The mean percentage distribution of arsenic species over the sum of inorganic and methylated species was 10.6% for inorganic arsenic, 18.4% for MA, and 70.9% for DMA. The intraclass correlation coefficient for three repeated arsenic measurements over a 10-year period was 0.80 for the sum of inorganic and methylated species and 0.64, 0.80, and 0.77 for percent inorganic arsenic, percent MA, and percent DMA, respectively.

**Conclusions:**

This study found low to moderate inorganic arsenic exposure and confirmed long-term constancy in arsenic exposure and urine excretion patterns in American Indians from three U.S. regions over a 10-year period. Our findings support the feasibility of analyzing arsenic species in large population-based studies with stored urine samples.

Inorganic arsenic (arsenite, arsenate) is a naturally occurring toxicant and carcinogen (International Agency for Research on Cancer 2004; National Toxicology Program 2002) that contaminates groundwater supply systems in countries around the world (Smedley and Kinniburgh 2002). In the United States, arsenic levels in drinking water > 10 μg/L—the U.S. Environmental Protection Agency (EPA) maximum contaminant level—disproportionately affect small communities in the West, Midwest, and Northeast regions (Focazio et al. 2000; U.S. EPA 2001). Flour and rice also contain inorganic arsenic, particularly if grown or cooked in areas with arsenic contamination in soil and water (Del Razo et al. 2002). The metabolism of inorganic arsenic in the human body results in methylarsonate (MA) and dimethylarsinate (DMA), which are excreted in urine together with unchanged inorganic arsenic (Aposhian and Aposhian 2006; Cullen and Reimer 1989). Seafood is a source of organic arsenic compounds (arsenobetaine, arsenosugars, arsenolipids) that have no or low toxicity compared with inorganic arsenic (Francesconi and Kuehnelt 2004).

In populations with low seafood intake, total urine arsenic and the sum of inorganic arsenic and methylated (MA and DMA) urine arsenic species are established biomarkers that integrate inorganic arsenic exposure from multiple sources (Calderon et al. 1999; Francesconi and Kuehnelt 2004; Hughes 2006; National Research Council 1999). The proportion of arsenic species that is excreted as inorganic arsenic, MA, or DMA also provides information on the metabolism of inorganic arsenic in the human body. The arsenic species excretion pattern in human urine is approximately 10–20% inorganic arsenic, 10–20% MA, and 60–80% DMA, with substantial variation among individuals (Chiou et al. 1997; Del Razo et al. 1997; Hopenhayn-Rich et al. 1996b; Vahter 2000). Individual arsenic excretion patterns, on the other hand, were fairly constant over time in studies of up to 1 year of follow-up (Concha et al. 2002; Steinmaus et al. 2005b). Because a higher proportion of MA in urine has been associated with an increased risk of cancer (Chen et al. 2003a, 2003b, 2005; Hsueh et al. 1997; Maki-Paakkanen et al. 1998; Steinmaus et al. 2006; Yu et al. 2000) and cardiovascular (Tseng et al. 2005; Wu et al. 2006) outcomes, there is substantial interest in characterizing the long-term arsenic species excretion patterns, especially for MA.

The objective of this study was to conduct an initial assessment of arsenic exposure and excretion pattern, as measured by total urine arsenic and urine arsenic species, in American Indians from Arizona, Oklahoma, and North and South Dakota who participated in the Strong Heart Study, a population-based prospective cohort study funded by the National Heart, Lung, and Blood Institute (Lee et al. 1990; Strong Heart Study 2008). On the basis of arsenic concentrations measured in public drinking water systems in the Strong Heart Study communities, we expected arsenic exposure levels to be higher in Arizona, intermediate in the Dakotas, and lower in Oklahoma. Given dietary patterns in the study communities (Stang et al. 2005), we expected arsenobetaine concentrations, a marker of seafood arsenicals, to be very low. Using three urine samples for each participant, we also assessed arsenic exposure and excretion patterns over a 10-year period and the feasibility of measuring arsenic species in long-term stored urine samples frozen at − 70°C.

## Materials and Methods

### Study population

The Strong Heart Study was designed to investigate cardiovascular disease and its risk factors in 13 American Indian communities located in Arizona, Oklahoma, and North and South Dakota (Howard et al. 1999; Lee et al. 1990). From 1989 to 1991, enrolled tribal members 45–74 years of age who were living in those communities were invited to participate. The aim was to recruit approximately 1,500 participants per region. In Arizona and Oklahoma, every eligible person was invited to participate, in person or by letter. In North and South Dakota, a cluster sampling technique was used (Lee et al. 1990). A total of 4,549 participants were recruited in 1989–1991 for a baseline visit and invited to participate in subsequent visits in 1993–1994 and 1998–1999. The participation rates were 62% at baseline and 89% and 88% for surviving participants at visits 2 and 3, respectively. All study visits included a questionnaire, a physical examination, and biological specimen collection. The Strong Heart Study protocol and consent form were approved by the Institutional Indian Health Service Review Boards and by the participating Indian communities. Informed consent was obtained from all participants. For the present study, we randomly selected 60 Strong Heart Study participants (20 from Arizona, 20 from Oklahoma, and 20 from the Dakotas) among those for whom we had stored urine samples from the three study visits (*n* = 3,197). Participants’ characteristics (age, sex, body mass index, smoking, and alcohol use) in the present study were comparable to the overall study population (Table 1).

### Arsenic determinations

Spot urine samples were collected in polypropylene tubes, frozen within 1–2 hr of collection, shipped in dry ice, and stored from 8 to 18 years at − 70°C in the Penn Medical Laboratory, MedStar Research Institute (Washington, DC, USA). The freezers have been operating under a strict quality control system to guarantee secure sample storage. For arsenic analyses, urine samples were thawed in August 2007, and up to 1.0 mL was transferred to a small vial, transported on dry ice to the Trace Element Laboratory, Graz University, Austria, and stored at − 80°C until analysis. Urine samples were frozen for an average of 13 years (range, 8–18 years) before analysis.

We measured total urine arsenic concentrations (expressed on an elemental basis) using inductively coupled plasma/mass spectrometry (ICPMS). The limit of quantification for total urine arsenic was 0.1 μg/L. We checked measurement accuracy with 1+19 diluted human urine no. 18 from Japan’s National Institute of Environmental Studies (Tsukuba, Japan) (e.g., 0.5 mL urine + 9.5 mL water). The measured mean (± SD) total arsenic concentration of 144 ± 4 μg/L (*n* = 19) was in agreement with the certified concentration of 137 ± 11 μg/L. Total arsenic concentrations exceeded the limit of quantification in all samples.

Urine concentrations of arsenite, arsenate, MA, and DMA (expressed on an elemental basis) were measured using high-performance liquid chromatography/vapor generation ICPMS (Lindberg et al. 2006). The limits of quantification were 0.1 μg/L for arsenite and 0.5 μg/L for arsenate, MA, and DMA. Arsenite and arsenate were below the limit of quantification in 2 (1%) and 126 (70%) samples, respectively. MA and DMA exceeded the limit of quantification in all samples. The interassay coefficients of variation for an in-house reference urine sample for arsenite, arsenate, MA, and DMA were 3.8%, 4.5%, 4.3%, and 1.9%, respectively (*n* = 18). We did not detect thio-DMA, an arsenic species that has been related to arsenosugar exposure (Hansen et al. 2003; Raml et al. 2005) and to high arsenic exposure in Bangladesh (Raml et al. 2007), in any of the study samples.

We measured urine arsenobetaine concentrations using cation-exchange chromatography on a Zorbax 300 SCX column (4.6 mm inner diameter × 250 mm; Agilent, Waldbronn, Germany) operated at 30°C. The mobile phase was 10 mM pyridine (pH 2.3, adjusted with formic acid) at a flow rate of 1.5 mL/min. We injected 20 μL of sample. The limit of quantification for urine arsenobetaine was 0.5 μg/L. We checked the accuracy of the measurements with 1+9 diluted human urine no. 18. The mean (± SD) measured value for arsenobetaine was 68 ± 2 μg/L (*n* = 18), in agreement with the certified concentration of 69 ± 12. Ninety-six (53%) samples had concentrations below the limit of quantification, reflecting infrequent seafood intake in the study population. The median (10th–90th percentiles) of urine arsenobetaine was 0.5 μg/L creatinine (< 0.5–137) [0.5 μg/g (< 0.5–6.1)]. Urine arsenobetaine concentrations were similarly low by region and other participant characteristics (data not shown).

### Other variables

Baseline information on sociodemographic data (age, sex, study region), smoking history, alcohol use, and body mass index (kilograms per square meter) were obtained from the Strong Heart Study questionnaires and physical examinations (Howard et al. 1999; Lee et al. 1990). Urine creatinine was measured at the laboratory of the National Institute of Diabetes and Digestive and Kidney Diseases Epidemiology and Clinical Research Branch (Phoenix, AZ, USA) by an automated alkaline picrate methodology run on a rapid flow analyzer (Lee et al. 1990). To account for urine dilution in spot urine samples, we divided urine arsenic (micrograms per liter) by urine creatinine (grams per liter) and expressed the concentrations of total urine arsenic and its species in terms of micrograms per gram creatinine.

### Statistical analyses

Because the limit of quantification was different for arsenite and arsenate and 70% of participants had arsenate concentrations below the limit of quantification, we used a bivariate Tobit multilevel model (Tobin 1958) to impute these values in participants with arsenite or arsenate levels below the limit of quantification. The bivariate Tobit multilevel model had random participant and region-specific intercepts and used total urine arsenic concentrations as an explanatory variable. Arsenite and arsenate concentrations in participants with values below the limit of quantification were imputed as the median of the participant-specific posterior distribution derived from the bivariate Tobit multilevel model. For arsenite, the posterior distribution median was 0.08 μg/L for each of the two samples below the limit of quantification. For arsenate, the median (10th–90th percentiles) of the posterior distribution medians for the 126 urine samples below the limit of quantification was 0.13 μg/L (0.07–0.26). We calculated urine inorganic arsenic concentration as the sum of arsenite and arsenate concentrations.

Because urine concentrations of total arsenic and its species were right-skewed, we used medians and 10th and 90th percentiles to describe their distributions. To evaluate variation in arsenic excretion patterns, we calculated the percentages of inorganic arsenic, MA, and DMA over the sum of inorganic and methylated species (%iAs, %MA, and %DMA). These percentages were normally distributed; we describe them using means ± SDs.

To evaluate the determinants of biomarkers of inorganic arsenic exposure at baseline and their trajectories over time, we used mixed linear models for change for log-transformed total arsenic, inorganic arsenic, MA, and DMA concentrations in urine. We used study region, sex, age, body mass index, smoking status, and alcohol consumption as fixed effects (explanatory variables). From these models, we estimated the ratios of the geometric means of baseline urine arsenic concentrations and of their annual rates of change comparing different categories of the explanatory variables. In sensitivity analyses, we also ran generalized estimated equation (GEE) models (Liang and Zeger 1986), overall and stratified by region. Findings were similar (data not shown).

We also used mixed linear models for change to model the %MA over the sum of inorganic and methylated arsenic species and to estimate the mean differences in baseline %MA and in its annual rate of change comparing different categories of study region, sex, age, body mass index, smoking status, and alcohol use. In sensitivity analyses, we also ran GEE models (Liang and Zeger 1986), overall and stratified by region. Findings were similar (data not shown).

We calculated intraclass correlation coefficients (ICCs) to describe the extent of within-individual compared with between-individual variability over time. A high ICC (close to 1) indicates that the within-individual variability is relatively small compared with the between-individual variability and that biomarkers of arsenic exposure and metabolism are relatively stable for an individual compared with between-individual variability. We performed all statistical analyses using the program R (R Project for Statistical Computing, Vienna, Austria).

## Results

### Participant characteristics

The mean (± SD) age of study participants was 54 ± 8 years, and 43% were men, with small differences across regions (Table 1). Similar to the overall Strong Heart Study population, participants from Arizona had higher body mass index compared with participants from Oklahoma and the Dakotas, and participants from the Dakotas were more likely to be current smokers and to use alcohol compared with participants from Arizona and Oklahoma.

### Arsenic concentrations

**Figure 1:**
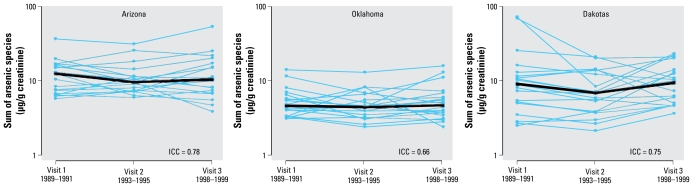
Sum of inorganic (arsenite and arsenate) and methylated (MA and DMA) species (μg/g creatinine) by study visit and region: (*A*) Arizona, (*B*) Oklahoma, (*C*) Dakotas. Blue lines connect the levels for each participant; black lines represent the median for each region.

Median (10th–90th percentiles) concentrations for total arsenic and for the sum of inorganic and methylated arsenic species at baseline (visit 1) were 10.5 μg/g creatinine (4.0–27.8) [12.5 μg/L (4.3–42.7)] and 7.2 μg/g (3.1–16.9) [9.8 μg/L (2.8–28.9)], respectively (Table 2). By region (Table 3, Figure 1), the median sum of inorganic and methylated arsenic species was higher in Arizona (12.5 μg/g), intermediate in the Dakotas (9.1 μg/g), and lower in Oklahoma (4.4 μg/g). After adjustment for sex, age, body mass index, smoking, and alcohol use, participants in Oklahoma and the Dakotas had approximately 80% and 29% lower urine concentrations, respectively, for the sum of inorganic and methylated arsenic species compared with participants in Arizona (Table 4), although the differences were statistically significant only for Oklahoma compared with Arizona. After multivariable adjustment, study region was the only statistically significant determinant of the sum of inorganic and methylated species at baseline (Table 4).

The sum of inorganic and methylated arsenic species remained relatively similar over 10 years of follow-up (Table 2, Figure 1). The ICC for the sum of inorganic and methylated species was 0.80, with little difference across regions (Figure 1). After multivariable adjustment, the average annual ratio over the 10-year follow-up was 1.23 [95% confidence interval (CI), 0.92–1.64], with no difference by participant characteristics (data not shown).

### Arsenic excretion pattern

**Figure 2:**
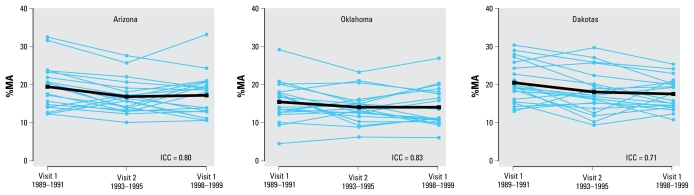
Percentage of MA over the sum of inorganic and methylated species by study visit and region: (*A*) Arizona, (*B*) Oklahoma, (*C*) Dakotas. Blue lines connect the levels for each participant; black lines represent the mean for each region.

The means (± SDs) for %iAs, %MA, and %DMA over the sum of inorganic and methylated species at baseline (visit 1) were 10.6 ± 5.2%, 18.4 ± 5.4%, and 70.9 ± 10.0%, respectively (Table 1). By region (Table 3, Figure 2), the mean %MA over the sum of inorganic and methylated arsenic species was lower in Oklahoma (15.4%) compared with Arizona (19.4%) and the Dakotas (20.5%). After multivariable adjustment, mean %MA was 6.0% (95% CI, 3.8–8.2%) point increased in men compared with women, and − 3.4% (95% CI, − 6.1 to − 0.7%) point decreased in participants in Oklahoma compared with Arizona (Table 5).

The percentage distribution of arsenic species remained relatively similar over 10 years of follow-up (Table 2, Figure 2). The overall ICCs for %iAs, %MA, and %DMA were 0.64, 0.80, and 0.77, respectively, with little difference across regions. After multivariable adjustment, the average annual change over the 10-year follow-up in %MA over the sum of inorganic and methylated species was − 0.7% (95% CI, − 1.5 to 0.2%), with no difference by participant characteristic (data not shown).

## Discussion

This study quantified for the first time individual biomarkers of arsenic exposure and metabolism, as assessed in urine, in American Indian populations from Arizona, Oklahoma, and the Dakotas over a 10-year period. Total urine arsenic and the sum of inorganic and methylated arsenic species concentrations were higher in Arizona, intermediate (but high in some samples) in the Dakotas, and lower in Oklahoma, as expected based on data on arsenic in groundwater (Focazio et al. 2000). For the sum of inorganic and methylated compounds, four samples (two in Arizona and two in the Dakotas) exceeded 35 μg/g creatinine, a safety standard used to monitor inorganic arsenic exposure in occupational settings (American Conference of Governmental Industrial Hygienists 2004). Arsenobetaine concentrations, related to seafood intake, were low compared with those in the U.S. general population (Caldwell et al. 2009), reflecting low seafood intake in the study communities. The constancy in urine arsenic concentrations in three urine samples collected over a 10-year period (1989–1999) is consistent with previous studies measuring arsenic in private and public drinking water systems over long periods of time (Karagas et al. 2001; Ryan et al. 2000; Steinmaus et al. 2005c) and support the observation that arsenic levels in drinking water are stable in the absence of public health interventions or changes in water sources. Our data also demonstrate the feasibility of using long-term stored urine samples for determining arsenic species in population-based studies.

The three Strong Heart Study visits were conducted between 1989 and 1999, before the U.S. EPA established the new arsenic maximum contaminant level in drinking water (10 μg/L). Data from public water systems in the states where the Strong Heart Study was conducted show that arsenic was higher in Arizona (county-level 95th percentile, 20.5 μg/L) compared with Oklahoma (county-level 95th percentile, 2.0 μg/L) and North and South Dakota (county-level 95th percentile, 4.0 μg/L) (Focazio et al. 2000). In the Strong Heart Study communities, arsenic concentrations measured in public drinking water systems in the 1990s and 2000s ranged from < 10 to 61 μg/L in Arizona and from < 1 to > 21 μg/L in North and South Dakota (no data are available for the Oklahoma communities, but levels were expected to be < 10 μg/L). Many participants in the Strong Heart Study rely upon private wells for water. Private wells do not need to comply with the U.S. EPA arsenic standard, and the water arsenic concentrations in many of them exceed 10 μg/L, particularly in the western states (George et al. 2006; Steinmaus et al. 2005c).

Urine arsenic concentrations in participants from Oklahoma were comparable with those for a representative sample of U.S. adults (median total urine arsenic in participants with arsenobetaine below the limit of detection, 3.4 μg/L) (Navas-Acien et al. 2008), reflecting generally low water arsenic concentrations (< 10 μg/L) in the United States. Urine arsenic concentrations for participants from the Dakotas and in particular from Arizona were substantially higher, comparable to urine concentrations in populations in Nevada, California (Steinmaus et al. 2005a), and Central Europe (Lindberg et al. 2006) exposed to moderately high arsenic in drinking water (~ 10 to > 50 μg/L) but much lower than urine concentrations in populations in Taiwan (Hsueh et al. 2003), Chile (Hopenhayn-Rich et al. 1996a), or Bangladesh (Chen et al. 2007; Kile et al. 2009), where drinking-water arsenic concentrations often exceeds 100 μg/L.

Because arsenic metabolism is thought to play a major role in inorganic arsenic toxicity, there is substantial interest in understanding the distribution and main determinants of arsenic species in urine. The distribution and interindividual variability of urine arsenic species in Strong Heart Study participants were comparable with those reported in other human populations (Chiou et al. 1997; Del Razo et al. 1997; Hopenhayn-Rich et al. 1996b; Vahter 2000). Studies conducted in Argentina (Concha et al. 2002) and Chile (Steinmaus et al. 2005b) previously showed a fairly constant within-person urine arsenic excretion pattern up to 1 year of follow-up. In our study, we extend this observation to 10 years, providing additional support for genetic factors influencing interindividual differences in arsenic metabolism (Hopenhayn-Rich et al. 1996a; Loffredo et al. 2003; Vahter 2000, 2002). Contrary to our study in the United States and previous studies in Argentina (Concha et al. 2002) and Chile (Steinmaus et al. 2005b), a recent study from Bangladesh measured low within-person reproducibility for %MA over a 2-year period (Kile et al. 2009).

Similar to studies in the United States (Steinmaus et al. 2005a), Europe (Lindberg et al. 2006), Chile (Hopenhayn-Rich et al. 1996b), Taiwan (Hsueh et al. 2003), and Bangladesh (Kile et al. 2009; Lindberg et al. 2008), men in our study had higher %MA in urine than did women. Some studies have also reported unadjusted associations of smoking and alcohol with higher %MA in urine (Hopenhayn-Rich et al. 1996a; Hsueh et al. 2003). In our study, after adjustment for sex, age, region, and body mass index, smoking and alcohol were not statistically associated with higher %MA, although our sample size was small. After adjustment for sex and other variables, participants in the Dakotas had a lower %MA concentration in urine compared with participants in Arizona or Oklahoma. The genetic determinants and health implications of differences in urine arsenic excretion patterns across study regions require further investigation.

Strengths of this study include the availability of repeated urine samples and the quality of the study measures. The Strong Heart Study used a standardized protocol for the recruitment of study participants, interviews, physical examinations, and collection and storage of urine samples (Lee et al. 1990; Strong Heart Study 2008). Some limitations include the relatively small sample size, the use of spot urine samples, the relatively high limit of quantification for arsenate, the storage of urine samples for > 10 years, and the difficulty in directly evaluating the impact of time of freezer storage. The long-term stability of inorganic arsenic, MA, DMA, and arsenobetaine in stored urine samples has been evaluated for a 6- to 8-month period (Chen et al. 2002; Feldmann et al. 1999) and is further supported by the findings of our study. Arsenic species such as methylarsonite [MA(III)] and dimethylarsinite [DMA(III)], however, tend to quickly convert to their corresponding pentavalent forms (MA and DMA) (Raml et al. 2007; Steinmaus et al. 2005b) and could not be measured. Arsenite and arsenate generally interconvert in urine depending on handling and storage conditions, and the sum of the two is generally used to measure inorganic arsenic in urine. In our study, to deal with data missing due to a relatively high limit of quantification for arsenate, we imputed arsenite and arsenate concentrations below the limit of quantification using total arsenic as an explanatory variable. In an alternative approach, all arsenate could have been reduced to arsenite (or all arsenite could have been oxidized to arsenate) before laboratory analysis to provide a stable measure of inorganic arsenic in urine. Overall, although arsenic-related health outcomes could not be assessed because of the limited sample size, our study supports the feasibility of using long-term stored urine samples for measuring arsenic exposure and metabolism in population-based studies such as the Strong Heart Study.

Arsenic in drinking water continues to be a public health problem in the United States, affecting several millions of Americans who live in small communities and rural areas, partly because private wells are not covered by the U.S. EPA maximum contaminant level, but also because of insufficient financial resources and public health infrastructure to ensure compliance with the arsenic standard in small communities. The well-established carcinogenic effect of arsenic (International Agency for Research on Cancer 2004; National Toxicology Program 2002), and evidence indicating a potential role of arsenic on cardiovascular disease and diabetes (Navas-Acien et al. 2005, 2006; States et al. 2009; Yuan et al. 2007) highlight the importance of ensuring arsenic levels below the U.S. EPA maximum contaminant level in all water supply systems, including those serving small communities.

## Conclusions

This study found low to moderate inorganic arsenic exposure and long-term constancy in urine arsenic concentrations and excretion patterns among American Indians from Arizona, Oklahoma, and the Dakotas who participated in a large population-based prospective cohort study. In communities with stable environmental exposure to inorganic arsenic and with relatively low fish intake, our study showed that a single determination of urine arsenic and its species provides a useful biomarker of ongoing arsenic exposure and of individual arsenic metabolism. Additional research is needed to further characterize the genetic and environmental determinants of arsenic metabolism and the health impact of arsenic exposure and metabolism, including cardiovascular disease and diabetes, in populations exposed to low and moderate levels of inorganic arsenic in drinking water.

## Figures and Tables

**Table 1:** Strong Heart Study participants’ characteristics at baseline.

	Arizona			Oklahoma			Dakotas		
Characteristic	Overall (*n* = 1,500)	Present study (*n* = 20)	*p*-Value^a^	Overall (*n* = 1,527)	Present study (*n* = 20)	*p*-Value^a^	Overall (*n* = 1,522)	Present study (*n* = 20)	*p*-Value^a^
Age [years (mean ± SD)]	56 ± 8	52 ± 8	0.03	57 ± 8	55 ± 8	0.58	56 ± 8	54 ± 6	0.23
Male (%)	36	45	0.48	42	35	0.65	43	50	0.65
Body mass index [kg/m^2^ (mean ± SD)]	32 ± 7	35 ± 6	0.06	31 ± 6	31 ± 7	1.00	29 ± 6	30 ± 4	0.46
Current cigarette smoking (%)	19	30	0.21	34	25	0.48	48	60	0.37
Current alcohol use (%)	40	35	0.82	37	45	0.49	48	60	0.37

a Based on Student’s *t*-test and Fisher’s exact test for means and percentages, respectively.

**Table 2:** Urine arsenic concentration (total and species) and percentage of urine arsenic species over the sum of inorganic and methylated species (∑As).

Measure	Visit 1 (1989–1991) (*n* = 60)	Visit 2 (1993–1995) (*n* = 60)	Visit 3 (1998–1999) (*n* = 60)
Urine concentration (μg/g creatinine) [median (10th, 90th percentiles)]			
Total arsenic	10.5 (4.0–27.8)	8.6 (4.0–18.0)	8.9 (4.2–26.7)
∑As (iAs + MA + DMA)	7.2 (3.1–16.9)	6.7 (2.9–14.4)	7.1 (3.5–20.4)
iAs (arsenite + arsenate)	0.6 (0.3–2.3)	0.6 (0.3–1.6)	0.6 (0.3–1.9)
MA	1.3 (0.5–3.6)	1.1 (0.5–2.0)	1.1 (0.5–3.3)
DMA	5.0 (2.2–12.8)	4.8 (2.2–11.4)	4.9 (2.7–15.0)
Arsenobetaine	0.5 (< 0.5–6.1)	0.5 (< 0.5–2.1)	< 0.5 (< 0.5–4.3)
Percentage of ∑As (mean ± SD)			
%iAs	10.6 ± 5.2	10.4 ± 4.6	8.9 ± 4.1
%MA	18.4 ± 5.8	16.3 ± 5.0	16.2 ± 5.1
%DMA	70.9 ± 10.0	73.4 ± 8.5	74.9 ± 8.0

iAs (arsenite, arsenate), inorganic arsenic.

**Table 3:** Baseline urine arsenic concentrations and excretion pattern by participant characteristics.

Characteristic	No.	Total arsenic (μg/g creatinine)^a^	∑As (μg/g creatinine)^a^	%iAs^b^	%MA^b^	%DMA^b^
Overall	60	10.5 (4.4–27.8)	7.2 (3.1–16.9)	10.6 ± 5.2	18.4 ± 5.8	70.9 ± 10.0
Region						
Arizona	20	16.9 (8.1–29.2)	12.5 (6.4–17.4)	12.4 ± 7.2	19.4 ± 5.8	68.3 ± 12.1
Oklahoma	20	6.4 (3.9–15.2)	4.4 (3.0–7.7)	8.6 ± 2.3	15.4 ± 5.2	76.0 ± 6.6
Dakotas	20	10.6 (3.8–48.1)	9.1 (2.7–30.2)	10.9 ± 4.3	20.5 ± 5.4	68.6 ± 12.1
Sex						
Female	34	10.6 (3.9–37.5)	6.6 (3.9–37.5)	8.4 ± 2.9	15.2 ± 3.9	76.3 ± 10.2
Male	26	10.1 (4.2–18.8)	7.8 (3.2–15.4)	13.4 ± 6.2	22.6 ± 5.1	64.0 ± 5.7
Age (years)						
45–54	35	11.3 (4.8–26.3)	7.5 (3.1–17.0)	11.7 ± 5.8	19.2 ± 6.1	69.1 ± 10.9
55–75	25	8.2 (4.0–24.4)	6.2 (3.2–14.9)	9.2 ± 3.7	17.3 ± 5.2	73.5 ± 8.1
Body mass index						
< 30 kg/m^2^	24	8.7 (4.2–18.6)	5.9 (3.2–15.4)	10.8 ± 5.6	19.9 ± 5.9	69.3 ± 10.5
≥ 30	36	12.3 (4.0–35.1)	7.8 (3.3–18.3)	10.5 ± 5.0	17.4 ± 5.5	72.1 ± 9.7
Smoking						
Never	18	8.6 (3.5–21.5)	4.6 (2.9–12.8)	8.5 ± 2.9	15.8 ± 5.2	75.7 ± 7.2
Former	19	13.9 (6.0–42.2)	11.5 (4.5–28.1)	9.9 ± 3.2	16.9 ± 4.8	73.2 ± 7.0
Current	23	10.5 (5.9–19.3)	7.5 (3.3–15.5)	12.9 ± 6.9	21.8 ± 5.5	65.4 ± 11.6
Alcohol						
Never	9	7.5 (3.5–16.1)	4.4 (2.9–9.2)	8.4 ± 2.6	14.6 ± 4.0	77.0 ± 6.0
Former	23	10.5 (5.7–38.7)	7.5 (4.0–19.2)	10.1 ± 3.8	18.2 ± 5.5	71.7 ± 8.5
Current	28	11.0 (4.2–23.3)	8.4 (3.2–16.4)	11.8 ± 6.4	19.8 ± 6.0	68.4 ± 11.5

Abbreviations: iAs, inorganic arsenic; ∑As, sum of inorganic arsenic, MA, and DMA. Urine arsenic excretion pattern is estimated as the percentage over the sum of iAs (arsenite, arsenate) and methylated species (MA, DMA).

a Median (10th–90th percentiles).

b Mean ± SD.

**Table 4:** Baseline ratio (95% CI) of geometric means for the sum of inorganic and methylated arsenic species in urine over time and by baseline participant characteristics.

Characteristic	No.	Urine arsenic geometric mean (95% CI)	Crude ratio (95% CI)	Adjusted ratio (95% CI)^a^
Region				
Arizona	20	11.2 (8.9–14.2)	1.00 (reference)	1.00 (reference)
Oklahoma	20	4.8 (3.9–5.8)	0.42 (0.28–0.63)	0.20 (0.07–0.55)
Dakotas	20	9.0 (5.7–14.0)	0.75 (0.50–1.12)	0.71 (0.25–2.04)
Sex				
Female	34	7.7 (5.8–10.2)	1.00 (reference)	1.00 (reference)
Male	26	8.0 (6.0–10.7)	1.03 (0.71–1.51)	0.79 (0.35–1.80)
Age (years)				
45–54	35	8.2 (6.5–10.3)	1.00 (reference)	1.00 (reference)
55–75	25	7.4 (5.2–10.6)	0.83 (0.57–1.22)	0.92 (0.39–2.20)
Body mass index				
< 30 kg/m^2^	24	6.7 (5.1–8.9)	1.00 (reference)	1.00 (reference)
≥ 30	36	8.7 (6.6–11.4)	1.23 (0.84–1.80)	1.58 (0.69–3.65)
Smoking				
Never	18	5.4 (4.0–7.3)	1.00 (reference)	1.00 (reference)
Former	19	10.8 (7.3–15.9)	1.99 (1.27–3.13)	2.69 (0.90–8.02)
Current	23	8.1 (5.9–11.0)	1.50 (0.97–2.31)	1.28 (0.39–4.22)
Alcohol				
Never	9	5.0 (3.3–7.5)	1.00 (reference)	1.00 (reference)
Former	23	8.7 (6.2–12.0)	1.80 (1.03–3.15)	1.60 (0.42–6.11)
Current	28	8.4 (6.2–11.3)	1.71 (0.98–2.99)	2.47 (0.65–9.30)

**Table 5:** Baseline difference (95% CI) in %MA over the sum of inorganic and mehtylated species.

Characteristic	No.	%MA (baseline mean ± SD)	Crude difference (95% CI)	Adjusted difference (95% CI)^a^
Region				
Arizona	20	19.4 ± 5.8	0.0 (reference)	0.0 (reference)
Oklahoma	20	15.4 ± 5.2	−3.5 (−6.8 to −0.3)	−3.4 (−6.1 to −0.7)
Dakotas	20	20.5 ± 5.4	1.3 (−1.9 to 4.5)	−0.3 (−3.1 to 2.5)
Sex				
Female	34	22.6 ± 5.1	0.0 (reference)	0.0 (reference)
Male	26	15.2 ± 3.9	6.9 (4.6 to 9.1)	6.0 (3.8 to 8.2)
Age (years)				
45–54	35	19.2 ± 6.1	0.0 (reference)	0.0 (reference)
55–75	25	17.3 ± 5.2	− 1.6 (− 4.4 to 1.2)	− 0.1 (− 2.5 to 2.2)
Body mass index				
<30 kg/m^2^	24	19.9 ± 5.9	0.0 (reference)	0.0 (reference)
≥ 30	36	17.4 ± 5.5	− 2.6 (− 5.4 to 0.2)	− 1.0 (− 3.2 to 1.3)
Smoking				
Never	18	15.8 ± 5.2	0.0 (reference)	0.0 (reference)
Former	19	16.9 ± 4.8	1.0 (− 2.3 to 4.2)	− 1.6 (− 4.5 to 1.3)
Current	23	21.8 ± 5.5	5.3 (2.2 to 8.5)	1.7 (− 1.5 to 4.9)
Alcohol				
Never	9	14.6 ± 4.0	0.0 (reference)	0.0 (reference)
Former	23	18.2 ± 5.5	2.8 (− 1.4 to 6.9)	0.0 (− 3.6 to 3.5)
Current	28	19.8 ± 6.0	4.7 (0.6 to 8.7)	0.4 (− 3.1 to 4.0)

a Adjusted for region, sex, age, body mass index, smoking, and alcohol.
